# The development of a framework of entrustable professional activities for the intern year in Ireland

**DOI:** 10.1186/s12909-020-02156-8

**Published:** 2020-08-18

**Authors:** Emily O’Dowd, Sinéad Lydon, Paul O’Connor, Josephine Boland, Gozie Offiah, Dara Byrne

**Affiliations:** 1 Discipline of General Practice, National University of Ireland Galway, Galway, Ireland; 2 Irish Centre for Applied Patient Safety and Simulation, National University of Ireland Galway, Galway, Ireland; 3 School of Medicine, National University of Ireland Galway, Galway, Ireland; 4 Medical Education Consultant, Galway, Ireland; 5 Royal College of Surgeons in Ireland, Dublin, Ireland

**Keywords:** Graduate medical education, Entrustable professional activities, Competency-based medical education, Medical education, Medical internship

## Abstract

**Background:**

Entrustable Professional Activities (EPAs) are units of professional practice that capture essential competencies in which trainees must become proficient before undertaking them independently. EPAs provide supervisors with a solid justification for delegating an activity to trainees. This study aimed to develop and ensure face validity of a set of EPAs for junior doctors in the first year of clinical practice in the Republic of Ireland.

**Methods:**

An iterative eight stage consensus building process was used to develop the set of EPAs. This process was based on international best practice recommendations for EPA development. A series of surveys and workshops with stakeholders was used to develop a framework of EPAs and associated competencies. An external stakeholder consultation survey was then conducted by the Irish Medical Council. The framework of EPAs was then benchmarked against the 13 core EPAs developed by the Association of American Medical Colleges (AAMC).

**Results:**

A framework of seven EPAs, and associated competencies resulted from this study. These EPAs address all core activities that junior doctors should be readily entrusted with at the end of the intern year, which is the first year of clinical practice in the Republic of Ireland. Each EPA contains a series of defined competencies. The final EPAs were found to be comparable to the AAMC core EPAs for entering residency.

**Conclusions:**

A framework of EPAs for interns in Ireland that are appropriate for the intern year has been developed by key stakeholders. The implementation of the EPAs in practice is the next step, and is likely to result in an improved intern training process and increased patient safety.

## Background

Evidence suggests that upon entering the workplace, junior doctors often lack the necessary skills to care for patients and feel unprepared for independent practice [1–6]. The experiences of junior doctors vary greatly depending on the settings within which they are placed [7, 8]. The quality of training and supervision trainees receive during their first year of practice varies between medical teams and hospitals, and likely contributes to the high level of stress and burnout in junior doctors in the Republic of Ireland [9, 10], and internationally [11, 12]. It is also important to acknowledge that junior doctors do not all necessarily acquire the requisite skills to care for patients at the same pace [13]. However, in the Republic of Ireland, as is generally the case internationally, the early years of training are based upon a time-based apprenticeship model. Under this model, junior doctors move to the next stage of training based upon the time they have spent in the role, and not on whether they have developed the necessary competencies to advance. A potential approach to address this issue would be to move away from the primarily time-based model, and adopt a Competency Based Medical Education (CBME) framework, utilising Entrustable Professional Activities (EPAs) to provide documented evidence for skill acquisition.

EPAs are defined as units of professional practice [14]. They describe discrete activities which can be entrusted to trainees, are essential to the profession, and encapsulate one or more core competencies [15]. They are observable, measurable, and executable within a specific time frame [16]. EPAs use observable work descriptors (e.g., ‘manage an outpatient clinic’) as opposed to person-descriptors (e.g., ‘good communicator’) that characterise other frameworks of competencies and milestones in medical education and training [15]. Therefore, EPAs are considered more readily implementable and appropriate than other systems of competency-based medical education. The purpose of EPAs is to formalise entrustment, and to provide supervisors with a solid justification for delegating an activity to trainees, at different levels of ability [14]. Levels of entrustment of trainees can vary from ‘entrusted to observe a senior colleague conducting the activity’ (Entrustment level 1), to ‘entrusted to supervise junior trainees’ (Entrustment level 5). Entrustment level 4, ‘performing the activity independently, without direct supervision’, is regarded as the threshold for competent independent practice [15].

EPAs have purported benefits for both trainees and their patients, as they represent a more systematic measure of physician competency than current practices [14]. This benefits the safety of patients by guaranteeing the competency of a physician completing a specific activity, and also ensures trainees are not put in situations that they are not competent to handle safely without supervision [16]. EPAs were developed with the intention of bridging the gap between theoretical competencies and practical clinical work [17]. Further, given that EPAs are being developed and implemented in Graduate Medical Education (GME) internationally [18], and in Undergraduate Medical Education (UME) [19, 20], they were considered a suitable method of implementing CBME in the Irish context. In the Republic of Ireland, there has been work done in the development of CBME and EPA frameworks in postgraduate specialist training for anaesthesiology and radiology [21]. However, EPAs have not been developed for the Irish intern year. Based on the emerging evidence from international use of EPAs, which indicates that they are acceptable to faculty and learners, and are useful for justifying entrustment [22], it was decided to develop EPAs specifically for the first year of clinical training for junior doctors in Ireland.

This paper describes the iterative, multi-step process used to develop a framework of core EPAs for interns in Ireland and the framework of EPAs that resulted from this process. The EPAs for interns in Ireland are intended to capture the core tasks which a doctor in the first year of training is required to be able to complete independently prior to progression to higher training. This study aims to explore the use of a multi-step stakeholder consensus building process to develop EPAs for the first year of clinical practice in the Republic of Ireland.

## Methods

### Aim

This study aimed to develop, outline and ensure face validity of a set of EPAs for interns in Ireland.

### Design

This study involved a mixed method stakeholder consensus building process, as has been used in other CBME studies [23, 24]. This approach triangulated data from multiple stages in a pragmatic development process. Both qualitative and quantitative data were generated and analysed.

### Setting and context

This study was conducted in the context of the Irish intern year, across the six national intern training networks. Following graduation from medical school in Ireland, each new graduate must complete an internship. Internship is the first year of clinical practice for junior doctors in Ireland, and an extension of medical school following graduation. The Irish intern year is comparable to the first year of residency under the United States system, or the Foundation Year 1 in the UK [25]. It is a pivotal year in medical training in Ireland, with interns experiencing clinical practice for the first time following graduation. At the conclusion of the year each intern receives a Certificate of Experience from the regulatory body, The Irish Medical Council, in recognition of their readiness to progress to further training.

### Ethical approval

Ethical approval for this study was received from the Chairperson of the Galway University Hospitals’ Clinical Research Ethics Committee.

### Analysis

Feedback from the different stages presented below was processed using a mixed-methods approach. Notes taken during group discussions in the workshops were collated, with group consensus recorded by the researchers. Qualitative responses to the surveys were reviewed by the research team and data from these fed into later stages. Percentage agreement of stakeholders was calculated.

### EPA development process

**Fig. 1:**
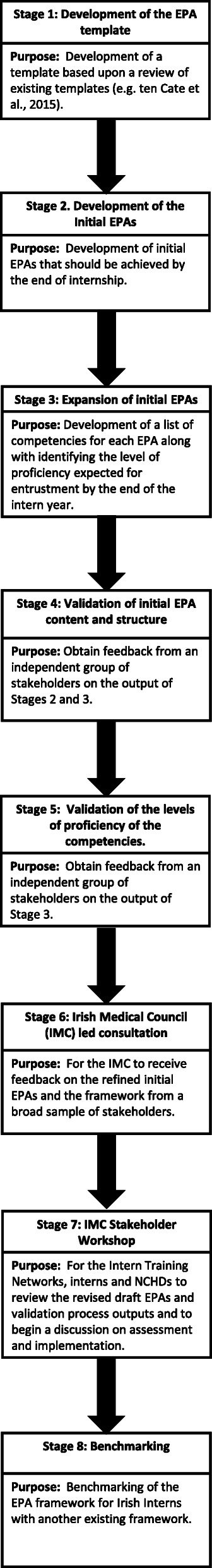
Stages of development process of EPAs for interns in Ireland

An overview of each of the stages in the consensus building process is shown in Fig. 1. This process followed Ten Cate and colleagues’ [26] broad guidelines for developing EPAs. These guidelines recommend: (1) identifying initial EPAs; (2) expanding the initial EPAs to include detailed descriptions and associated competencies; and (3) validating the framework of EPAs and competencies using a variety of methods [26]. These three broad guidelines were operationalised by the research team. This process resulted in eight discrete stages in the consensus building process.

#### Stage 1: development of the EPA template

*Participants:* The research team, consisting of both medical educationalists and researchers with experience in medical education.

*Process:* The participants in stage 1 carried out a review of the literature on EPAs and identified key components of EPA templates as recommended by Ten Cate et al. [26]. This review was conducted with the aim of developing a bespoke EPA template for the Irish intern year.

#### Stage 2: development of the initial EPAs

*Participants:* Participants were four interns at the end of their intern year, four other non- consultant hospital doctors (NCHDs) including three senior house officers (SHOs) and one specialist registrar, two hospital consultants, one chief academic officer, two intern training network coordinators, two medical educators, and one research methodologist. Participants were selected to represent a range of valuable and complementary perspectives on what should be expected at the end of internship.

*Process:* A half-day workshop was held in Galway, Ireland, in July 2015, for stakeholders to develop the initial EPAs. The workshop was facilitated by the research team and included presentations on the aims of this research. An iterative process which included ranking exercises, generating ideas through group discussion, and reaching agreement on the ideas through consensus was then used to develop a core set of initial EPAs.

#### Stage 3: expansion of initial EPAs

*Participants:* The same participants as in stage 2.

*Process:* The participants in this stage were subdivided into small groups during the workshop to expand the EPAs. This involved identifying the associated competencies required to achieve each EPA, and deciding the level of proficiency at which each of the competencies within the EPAs should be scored. They were also tasked with drafting a narrative description of each EPA.

#### Stage 4: validation of initial EPA content and structure

*Participants:* Six NCHDs (two interns, two SHOs, two registrars) and three hospital consultants. These participants had not been involved in the early stages of the consensus building process. In addition to their clinical role, three of the participants in Stage 4 were also intern training network coordinators and one was an intern tutor. Participants were selected based on their years of experience, with a mix of both senior and junior physicians to provide a range of insights into the intern year.

*Process:* Stage 4 was the first round of a two-part online survey intended to provide feedback on the EPAs developed in previous stage (see stage 5 for a description of part 2 of the online survey). The survey was developed for this study, and can be found in Additional file 1. The purpose of stage 4 was to enhance the face validity of the initial EPAs by circulating them and their associated competencies to a wider group of stakeholders. The survey was accompanied by a short explainer video outlining the EPA framework and what was required of each respondent in order for them to provide feedback. Stakeholders provided data on the appropriateness of the EPAs and their associated competencies identified in Stage 3. The Stage 4 participants were asked whether they thought each of the initial nine EPAs was a key activity that interns should be able to perform by the end of the year. They were also asked if any of the EPAs or competencies should be eliminated, whether each of the competencies were appropriate for the EPAs to which they were linked, and if there was a need for additional EPAs or competencies. Agreement on these questions was calculated using percentages.

#### Stage 5: validation of the levels of proficiency of the competencies associated with the initial EPAs

*Participants:* A total of 10 doctors who had not participated in earlier stages completed the survey (Two interns, two SHOs, two registrars, two consultants). Participants were again selected to give a variety of insights into the intern year based on their years of clinical experience.

*Process:* Stage five was the second round of the two-part online stakeholder consultation intended to further enhance the validity of the EPAs. This survey was developed for the study, and detailed descriptions of the questions can be found in Additional File 2. The stakeholders in this stage were sent an online survey with an explanatory video, similarly to participants in stage 4. They were then asked to give feedback on the type (e.g., knowledge, clinical skills, attitudes/behaviours) and whether they agreed upon the level of proficiency required that had been assigned to the competencies associated with each initial EPA in earlier rounds. Agreement on the required proficiency level and types of competencies was calculated using percentages.

#### Stage 6: Irish medical council (IMC) led consultation

*Participants:* A total of 80 stakeholders who had not been involved in the development of the EPAs, consisting of representatives from regulators, postgraduate medical training bodies, medical educators, NCHDs, and hospital consultants. These participants were selected with the aim of providing a broad and varied insight into the EPAs, based on their experiences as educators, trainees, and regulators of professional standards.

*Process:* The refined draft EPAs resulting from stages 4 and 5, and EPA framework were sent to 80 stakeholders via an online survey in order to obtain broad feedback on the EPAs and their appropriateness for the Irish intern year and to invite further comment. This survey was developed specifically for use in this study, and the questions can be found in Additional File 3.

#### Stage 7: IMC stakeholder workshop

*Participants:* A total of 20 participants including seven interns, five intern trainers, four other NCHDs, two hospital consultants and two representatives from the Irish Medical Council.

*Process:* A facilitated workshop hosted by the IMC, facilitated by four members of the research team. Attendees were presented with the EPAs and EPA framework which resulted from stage 6, prior to the half-day workshop in June 2016. During the workshop, participants were presented with the context of the study and the work completed to date. They were asked to agree on the level required for entrustment on each EPA as a whole (rather than discriminating between individual competencies), and to edit or remove competencies that were not achievable at this level.

#### Stage 8: benchmarking

*Participants:* Members of the research team.

*Process:* The final stage of the consensus building process involved benchmarking the newly developed EPA framework for Irish interns against the AAMC core EPAs for entering residency and the associated Critical Competencies. Benchmarking occurred at two levels, comparing first the higher-order EPAs and then, at a more granular level, the associated competencies within the EPAs in each framework.

## Results

### Stage 1: development of the EPA template

The final agreed template is presented in Additional file 4. The template developed included space for the EPA title, the Irish Medical Council Domains of Good Professional Practice [27] that are relevant to the EPA, and the list of competencies that fall under the EPA. Competencies were categorised by type, which included: knowledge (prior knowledge required to perform the professional activity); skill (the clinical skills required to complete the professional activity); and “attitudes/behaviour” (the attitudes and behaviours associated with each professional competency- such as relating to patients, communication, collaboration, etc.).

### Stage 2: development of the initial EPAs

The workshop conducted as part of Stage 2 resulted in an initial list of nine EPAs structured around intern work and educational practices. These initial EPAs are presented in Table 1.

**Table 1:** Nine initial EPAs

Nine initial EPAs	
Admit a Patient	
Request and interpret investigations	
Perform Basic Procedural Skills	
Manage the work of in-patient care	
Prescribe and monitor drugs and fluid	
Recognise and manage the deteriorating/acutely unwell patient	
Transition and discharge patient care	
Engage in personal and professional development	
Identify compromises to patient care	

### Stage 3: expansion of initial EPAs

Following small group discussions and consensus being achieved within the wider group, between 11 and 18 competencies were outlined for each of the nine initial EPAs listed in Table 1. For the majority of the EPAs the entrustment was judged to be level 4 (The intern may perform an activity independently with mainly informal, indirect supervision), or level 5 (Intern may provide supervision and instruction to junior learners).

### Stage 4: validation of initial EPA content and structure

There were high levels of agreement between participants on the appropriateness of the EPAs and associated competencies in stage 4. The majority of EPAs had acceptable agreement on both of these aspects, however, the EPA “Transition and discharge patient care” was an exception. For this EPA, 90.9% of respondents agreed that it was a core activity for an intern, and participants agreed that only 54.5% of competencies within this EPA should be performed by an intern. More detail on the percentage agreement on each EPA can be found in Additional file 5.

A number of additional EPAs were also suggested by participants during this stage (e.g., “communicating with families”, “Work as part of a team” and “present and communicate within a team structure”). However, as they did not fit the definition of EPAs, they were not included as new EPAs. Qualitative comments were also collected regarding the EPAs and associated competencies developed in the earlier stages. For example, for EPA 3 ‘Perform basic procedural skills’, the participants’ comments were centred on whether or not interns should perform certain skills (e.g., “*should interns be able to do a lumbar puncture?*”; “*I don’t think interns should be instigating non-invasive ventilation without senior/anaesthetic input*”). Based on the qualitative feedback to this survey, some amendments were made to the initial EPAs, but no additional EPAs were added. The amendments made included adding some additional competencies and alterations to some terminology.

### Stage 5: validation of the levels of proficiency of the competencies associated with the initial EPAs

Agreement among stakeholders on the levels of proficiency required for each competency within the initial EPAs ranged from 70 to 100%. Where there were disagreements on proficiency ratings, these were highest for competencies within EPA 4 “Manage in-patient care” (70.0% agreement) and EPA 3 “Perform basic procedural skills” (72.7% agreement). Where participants disagreed, they were asked to indicate what they considered the appropriate level of proficiency would be. Despite minor disagreements, the agreement was considered acceptable and the proficiency levels remained the same. More details on level of agreement on proficiency ratings can be found in Additional File 5.

Participants also agreed on the classification of the types of competencies set in the previous stages (i.e. classifying competencies as knowledge, clinical skills, or attitudes/behaviour), with agreement ranging from 58.3% for EPA 1 “Admit a patient”, to 90%. While agreement was acceptable for these classifications, it was decided that this level of detail was not required for the EPA framework moving forward, and it was removed from the framework by the research team following this stage.

### Stage 6: IMC led consultation

Forty survey responses were obtained for the survey that was distributed in stage 6 (response rate = 50%). The IMC consultation survey resulted in the removal of EPA 8 (engage in personal and professional development) and EPA 9 (identify compromises to patient care) from the initial draft EPAs. The rationale for removing these two EPAs was that 10 and 7 respondents respectively did not think EPA 8 and 9 met the definition of an EPA. This resulted in seven EPAs. However, although initial EPA 8 was not considered as an EPA by definition, it was felt that ‘personal and professional development’ was important and so was included in the final framework as a set of related continuing professional development activities.

### Stage 7: IMC stakeholder workshop

The Stage 7 workshop resulted in agreement on the level required for entrustment for each of the seven EPAs. It was decided that it should be level 4 (Intern may perform an activity independently with mainly informal, indirect supervision) for all of the seven remaining EPAs (see Table 2 and Additional File 6 for a description). The definition of this level of entrustment in the contest of the intern year in Ireland was agreed upon as the supervisor being ‘ … on-site and available, just in case’. This level of entrustment is consistent with Ten Cate and colleagues’ recommendations [15].

**Table 2:** Intern Year EPAs, including titles and descriptions

No.	EPA Title	Description
1	Clerk a patient	- The doctor can clerk a patient in the outpatient and day care setting, admit a patient to the ward and has a good understanding of decision to admit criteria.
2	Request and interpret basic investigations	- The doctor can request appropriate, and interpret, basic diagnostic laboratory and radiological investigations.
3	Perform essential procedural skills	- The doctor is competent in the following essential procedures: o Hand hygiene o Venepuncture o Peripheral intravenous cannulation o Blood cultures from a peripheral vein o Arterial blood gas sampling o Electrocardiogram (ECG) o Nasogastric tube insertion o Urinary catheter insertion o Preparation, reconstitution, dilution and administration of iv drugs o Blood sampling and blood cultures from central line and tunnelled lines o Sterile field set up o Sterile glove application
4	Manage the work of in-patient care	- The doctor can manage their daily workload to prioritise, delegate tasks, advance patient flow, and deliver patient-centred care.
5	Prescribe and monitor drugs and fluids	- The doctor can prescribe safely, and in compliance with legal requirements, in both a hospital and community setting, and in an elective and emergency setting.
6	Recognise and manage the deteriorating/acutely unwell patient	- The doctor can identify and respond to the acutely unwell patient appropriately.
7	Handover and discharge a patient	- The doctor can handover and receive the handover of a clinical case to/from colleagues and manage the discharge of a patient competently.
	**Set of related activities**	**Description**
	Engage in Personal and Professional Development	- At the end of internship, the doctor has achieved all EPAs to level 4, and is a well-rounded professional who strives to improve themselves clinically and educationally.

### Stage 8: benchmarking

Benchmarking of the EPAs for interns in Ireland with the Association of American Medical Colleges (AAMC) Core EPAs for entering residency from the United States [28] was carried out in Stage 8. The benchmarking across EPAs is illustrated in Tables 3 and 4 presents the more detailed benchmarking of competencies across the two frameworks. The EPAs for interns in Ireland were found to align with the AAMC framework of EPAs, in terms of the content of the EPAs themselves, as they encompass similar requirements of the trainees. This can be seen in Table 3 below, where the darker shading indicates stronger alignment. For example, EPA 1 from the AAMC EPAs, “Gather a history and perform a physical evaluation” aligned strongly with EPA 1 from the Irish internship framework, “Clerk a patient”, which expects similar competencies from a trainee. It aligned to a weaker extent with EPA 4 “Manage in-patient care” and EPA 6 “Manage the acutely unwell/deteriorating patient”. Table 4 illustrates how one competency from the AAMC framework aligns with multiple competencies across all seven of the Irish intern EPAs, and with the supporting set of related professional development activities.

**Table 3:**
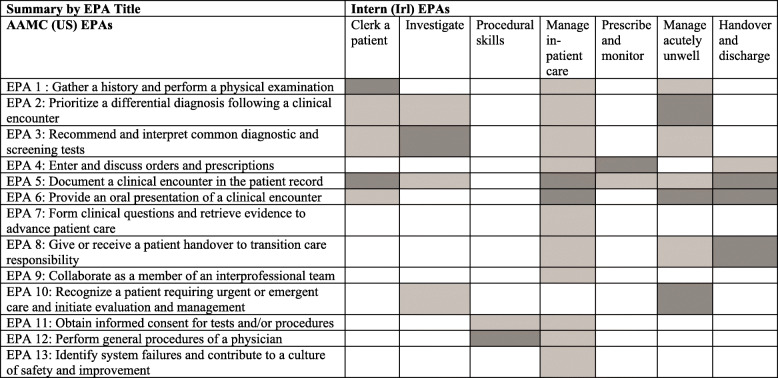
**Alignment between AAMC EPAs (US) and Intern EPAs (Irl)**

*Note*. Darker shading is indicative of stronger alignment

**Table 4:** Sample of the mapping of competencies between the AAMC EPAs (US) and Intern (IRL)EPAs

AAMC (US) EPA 1: Gather a history and perform a physical examination	Maps to …	Intern (IRL) EPA competencies
**AAMC Critical competency:** Gather essential and accurate information about patients and their conditions through history taking, physical examination, and the use of laboratory data, imaging, and other tests		EPA 1: Competencies 1, 2, 3, 4, 5, 7 EPA 2: Competency 1 EPA 4: Competency 1 EPA 5: Competency 2 EPA 6: Competencies 3, 4

## Discussion

The early years of medical training have traditionally been based upon a time-based apprenticeship model in the Republic of Ireland and internationally. Therefore, junior doctors advance to the next stage of training based upon the time they have spent in the role, and not on whether they have developed the necessary competencies. A potential approach to address this issue would be to move away from the time based model and adopt a CBME framework.

The decision to explore the use of EPAs as a CBME framework for structuring the intern year in Ireland was made based on the emerging evidence from international use of EPAs, which indicates that they are acceptable to faculty and learners, and useful for justifying entrustment [22]. EPAs are seen as a way of bridging the gap between the theoretical aspects of CBME and practical clinical work, by focusing on observable, measurable activities instead of person-based descriptors which are hard to assess [15]. The benefits to patients and trainees of ensuring each trainee is competent in a task prior to being entrusted to complete it are apparent [16]. Therefore, we decided that EPAs would be an appropriate framework with which to restructure the intern year in Ireland. Through an iterative process, seven EPAs, and a set of supporting professional development activities, were developed. These seven EPAs are intended to capture the level of performance that is expected at the end of the intern year. The EPAs are general in nature, and could be assessed and applied across different rotations within the intern year to determine trainees’ proficiency in the activities within different contexts. The EPAs, and their linked competencies, have been refined and explicated by key stakeholders, and with the inclusion of an assessment process, are suitable for piloting in clinical practice.

The consensus building process used to develop the EPAs for interns in Ireland was consistent with best practice for EPA development [14]. A recent systematic review [18] found that, of 39 EPA development papers included, only five papers used four or more steps (e.g., literature review, workgroup discussion, online survey) in their development process. Notably, studies that used more steps in the development process were often of a higher quality than those that used fewer [18]. With this in mind, the extensive process used to develop the framework of EPAs for interns in Ireland reported in this paper would appear to be of a high standard [18] when considered together with the extant work on EPAs in GME.

The framework of EPAs for interns in Ireland consisted of seven EPAs and was comparable to the 13 AAMC EPAs. These EPAs specifically address the requirements of the Irish intern year, and with the use of appropriate work-based assessment tools, aim to help interns achieve the necessary outcomes and skills by the end of the year. Although the AAMC EPAs are greater in number, they were found to be broadly comparable to the framework of EPAs for interns in Ireland, albeit at an undergraduate as opposed to graduate stage of medical training. The AAMC Core EPAs were developed for the point of entering the first year of residency, as opposed to the Irish intern EPAs, which are intended to be completed throughout the intern year which is broadly equivalent to the first year of residency. Benchmarking with the AAMC framework ensures that while the EPAs are focused on the Irish intern year, they are comparable, even considering different trainee stages and different requirements of level of independent clinical practice [25].

Benchmarking is an important exercise when developing medical education curricula, in order to ensure quality improvement takes place, and to give evidence for this improvement to stakeholders [29], and its deployment in this process ensures the quality, and sufficiency, of the intern year EPAs. Future research could benchmark the EPAs for interns in Ireland with other systems, for example the UK Foundation Professional Capabilities [30], to further explore the strength of the Irish framework in comparison to international standards. Ten Cate gives a general suggestion of between 20 and 30 EPAs for a postgraduate training course [31]. That paper also indicates that EPAs should be limited in number and address broad requirements of the training course [31]. As internship in Ireland is only 1 year of postgraduate training, as compared to the 2 years of foundation training in the UK, and longer programmes for specialty training in Ireland, and since the stakeholder process has indicated that the EPAs cover the broad requirements of internship, we make the case that seven EPAs are sufficient in this instance.

In order to fully capture the skills required of interns by the end of their year, the EPA framework was supplemented with a set of related continuing professional development activities (see Additional File 6). These activities were considered essential to intern education, however they were not necessarily discrete, observable work, and as such the stakeholders did not consider them to fit the definition of an EPA [15]. Future research should explore how best to operationalise these tasks in order to assess them using appropriate tools of work-based assessment.

### Recommendations

A number of recommendations logically follow on from the development of the framework of EPAs for interns in the Republic of Ireland. First, there is a need to evaluate the quality of EPAs developed. The method of development as reported in this paper was rigorous, however apart from face validity being determined by the stakeholders, no external quality assessment has been conducted on the EPAs. Applying a quality assessment tool such as the Quality of EPA (QUEPA) [32] or the EQual Quality Rubric for EPAs [33] to the developed EPAs would ensure the framework of EPAs is of a high quality, and in turn improve the standards by which trainees are measured. We recommend that the EPAs for interns in Ireland are examined and validated by experts in the field, using one of these quality assessment tools, prior to their implementation in the intern year.

Secondly, we recommend that the tools and methods used to assess trainees’ competence in the EPAs are further developed. Assessment and feedback are fundamental to CBME [34]. Small scale studies on implementing EPAs in practice have used a variety of tools and methods for assessment, including simulation, chart audits, and trainee portfolios [18]. It is also important to consider the resources and practicality of any proposed methods of assessment in the next phase of development. The current EPA template includes recommended observation and review tools, for the purpose of workplace-based assessment. These comprise five assessment methods; Case Presentation (CP), Direct Observation of Procedural Skills (DOPS), Case Based Discussion (CBD), Reflective Journal (RJ), and Team Review (TR), all of which were adapted from Boland and colleagues [35]. The proposed number and type of assessments to be completed for each EPA can be found in the EPA descriptions in Additional File 6. However, further expansion of these, along with an analysis of the resources required to use them, would be beneficial.

Finally, the implementation of these EPAs in practice must be explored. We recommend conducting a small-scale pilot or feasibility study of the implementation of these seven core EPAs and their associated assessment tools for the intern year, prior to their widespread introduction for interns. This pilot would identify issues with the EPAs that may emerge such as organising the systemic changes necessary to deliver the curriculum and work-based assessment methods to include faculty training, additional educational supervisors and supportive technology. A pilot could also help to adapt teaching and learning strategies, and change the culture of education so that CBME and EPAs are accepted [36]. Finally, it would provide an accurate estimation of the resources required for successful implementation. To date, there is little data available on piloting or implementing EPAs in a GME context [18].

### Limitations

There are a number of limitations of the current study that should be considered. First, the EPAs and associated assessment tools for interns in Ireland have not yet been implemented in practice. They have been developed without establishing the feasibility of their implementation in the clinical environment. Therefore it is unknown how they will be received by interns and supervisors. However, the involvement of some interns and supervisors in the development process augurs well for how they will be received by stakeholders on a larger scale. Developing EPAs without trialling them in a clinical environment is currently common practice [18], with few studies investigating their use in clinical practice – particularly the impact they may have on the workload of supervisors. Therefore, there is a clear need to evaluate the implementation of the intern year EPAs in clinical settings and to engage with stakeholders and key performance indicator data in order to explore their impact.

Secondly, while an iterative process was used to develop the EPAs, small numbers of participants were involved at each stage. Larger workshops could have changed the development of the EPAs by introducing other opinions. However, the participants that were involved were from a variety of different backgrounds and expertise levels, which provides unique insight on the EPAs, despite the small sample size. The scale of the sector and the geographic proximity of intern networks in Ireland also facilitated face-to-face workshops in a central location. The number of stakeholders involved was comparable to those reported in other EPA development papers [18].

A final limitation is that the EPAs were developed specifically for the Irish context. Developing country-specific EPAs could limit their generalisability to international systems and in turn hinder physicians who wish to work outside of Ireland. This, however, is typical practice among current reports of EPA development, with EPAs developed across several countries including The Netherlands, New Zealand, and Australia [18, 37]. The EPAs developed by these other countries were also designed to be implemented at different stages of medical training, compared to the Irish Intern EPAs [18, 37]. Further, as the intern year in the Republic of Ireland is unique in terms of structure and duration, and also custom and practice within Irish training hospitals, it was considered appropriate to develop a framework of EPAs that reflected the work of interns in Ireland, albeit aligned with the AAMC Core EPAs [25]. This benchmarking is particularly important due to the prevalence of physician migration [38]. Further benchmarking with the UK system, and testing of the Irish intern EPAs in other settings, could also support the relevance of the Irish EPAs in the context of international systems.

## Conclusions

After an iterative consensus building process, seven EPAs for interns in Ireland were developed. These EPAs and associated work-based assessment tools must be piloted in a clinical context to ensure that they are fit for purpose, and once introduced to the intern year, could support junior doctors to achieve competency in a manner that safeguards patient safety.

## Supplementary information

**Additional file 1.**

**Additional file 2.**

**Additional file 3.**

**Additional file 4.**

**Additional file 5.**

**Additional file 6.**

## Data Availability

The datasets used and/or analysed during the current study are available from the corresponding author on reasonable request.

## Abbreviations

AAMC: Association of American Medical Colleges
BMT: Basic Medical Training
CBD: Case Based Discussion
CBME: Competency Based Medical Education
CP: Case Presentation
DOPS: Direct Observation of Procedural Skills
EPA: Entrustable Professional Activity
EQual: Queens EPA Quality Rubric
GME: Graduate Medical Education
IMC: Irish Medical Council
QUEPA: Quality of EPA (tool)
RJ: Reflective Journal
TR: Team Review
UME: Undergraduate Medical Education
