# Supplementary material for: The development of a framework of entrustable professional activities for the intern year in Ireland
Source: BMC Med Educ. 2020 Aug 18;20:273. doi: 10.1186/s12909-020-02156-8 (PMC7433170; doi:10.1186/s12909-020-02156-8)
Supplement: Supplementary file 1 — Additional file 1. [file 12909_2020_2156_MOESM1_ESM.pdf]

EPAs

1. For each of the nine EPAs below, please indicate whether you regard it as a key activity which an intern should be able to perform independently by the end of training.

	Yes	No
Admit a patient	◯	◯
Request and interpret investigations	◯	◯
Perform basic procedural skills	◯	◯
Co-ordinate in-patient care	◯	◯
Prescribe and monitor drugs and fluid	◯	◯
Recognise and manage the deteriorating/acutely unwell patient,	◯	◯
Transition and discharge patient care	◯	◯
Engage in personal and professional development	◯	◯
Identify compromises to patient care	◯	◯

2. If you have answered no to any of the EPAs, please provide a short explanation below

3. Are there additional EPAs that you think should be included?

◯ No

◯ Yes, please add additional competencies to the box below

EPA 1: Admit a patient

DESCRIPTION: At the end of internship, the doctor should be able admit a patient electively and as an emergency in addition to making the decision to admit. They should be able to take a focused history, perform a thorough physical examination and identify pathological findings. This should form the basis for requesting laboratory and radiological investigations and consultations that are pertinent to the case, rationalised and reflect best practice. The admission note should be logically structured and prioritise diagnoses, interpret investigations and have a treatment plan.

4. For each competency, please indicate whether it is appropriate for this EPA.

	Appropriate	Inappropriate
Establish rapport with patient	◯	◯
Consider cultural and other influences that may affect the patient’s description of their symptoms	◯	◯
Obtain a history from other sources as required (collateral, own doctor, pharmacy)	◯	◯
Gather a focused history and perform a fluid and sequential clinical examination	◯	◯
Demonstrate respect for the patient’s privacy, dignity and culture	◯	◯
Recognise abnormal findings	◯	◯
Request laboratory and radiological investigations and consultations that are pertinent to the case and reflect best practice.	◯	◯
Act on conditions and presentations that require immediate intervention, instigate initial resuscitation/treatment and communicate with senior colleagues	◯	◯
Prioritise a differential diagnosis following a clinical encounter	◯	◯
Put a treatment and further management plan in place	◯	◯
Consent a patient according to Medical Council guidelines	◯	◯
Communicate transfer plan to healthcare receiver (ward, theatre, ICU) and patient	◯	◯
Inform consulting teams of the reason for referral and consultation if required	◯	◯
Document all findings clearly and legibly in the patient chart	◯	◯
Rationalise medications – commence, discontinue	◯	◯
Recognise patient at risk of deterioration	◯	◯
Communicate with senior colleagues and nursing staff to ensure all have a shared mental model of the patient’s condition and needs	◯	◯
Request laboratory and radiological investigations and consultations that are pertinent to the case and reflect best practice.	◯	◯
Act on conditions and presentations that require immediate intervention, instigate initial resuscitation/treatment and communicate with senior colleagues	◯	◯
Prioritise a differential diagnosis following a clinical encounter	◯	◯
Put a treatment and further management plan in place	◯	◯
Consent a patient according to MC guidelines	◯	◯
Communicate transfer plan to healthcare receiver (ward, theatre, ICU) and patient	◯	◯
Document all findings clearly and legibly in the patient chart	◯	◯
Rationalise medications – commence, discontinue	◯	◯
Recognise patient at risk of deterioration	◯	◯
Communicate with senior colleagues and nursing staff to ensure all have a shared mental model of the patient’s condition and needs	◯	◯

5. If you thought any of the competencies were inappropriate, please provide some short explanation below.

6. Are there additional competencies that you think should be included?

- ◯ No
- ◯ Yes, please add additional competencies to the box below

EPA 2: Request and interpret investigations

DESCRIPTION:At the end of internship, the doctor should be able to request the most appropriate and interpret basic diagnostic laboratory and radiological investigations. They should be able to rationalize the investigations to the patient and medical team and communicate the indications to the relevant testing labs and departments.
The interpretation of these tests should reflect consideration for the patient’s demographics and lead to formulation of a most likely diagnosis and prioritize the differentials. They should be able to compile the results of the investigations so as to commence a treatment plan or identify the need for further investigations.

7. For each competency, please indicate whether it is appropriate for this EPA.

	Appropriate	Inappropriate
Make informed decisions to request investigations and screening tests based on patient information and best practice	○	○
Counsel and educate patients so they can be involved in the decision making process	○	○
Correctly identify the patient and patient details so as to prevent error	○	○
Incorporate an awareness of cost-effectiveness, risk-benefit analysis into their decision making	○	○
Demonstrate an analytical approach to clinical situations	○	○
Communicate the clinical situation and rationale for the investigation to the laboratory, radiology or other departments	○	○
Actively seek the result of the investigation – follow through on the request	○	○
Interpret the results of basic radiology reports and images (plain films) and basic laboratory reports required for the area of practice (haematology, biochemistry, microbiology)	○	○
Record the results of investigations in patient notes and the implications of the results in the patient’s further care and management plan	○	○
Communicate the results and their interpretation to the medical team and patient	○	○
Make recommendations for the need for further investigations or screening	○	○
Act immediately on an abnormal result and seek assistance as appropriate	○	○

8. If you thought any competencies were inappropriate, please provide some short explanation below

9. Are there additional competencies that you think should be included?

- ◯ No
- ◯ Yes, please add additional competencies to the box below

EPA 3: Perform basic procedural skills

DESCRIPTION: By the end of internship, the doctor should be confident and practiced in performing basic procedural skills.

10. For each competency, please indicate whether it is appropriate for this EPA.

	Appropriate	Inappropriate
Decide when a procedural skill is necessary	◯	◯
Demonstrate adaptability in using different systems and equipment brands to perform a procedure	◯	◯
Recognise one’s own limitations in complex patient cases and call for help	◯	◯
Perform 5 moments of hand hygiene	◯	◯
Obtain verbal consent from the patient by explaining the indications and complications of the procedure	◯	◯
Put the patient at ease	◯	◯
Perform the procedure to safe standard	◯	◯
Can set up a sterile field, maintain aseptic and sterile conditions	◯	◯
Recognise any immediate failures or complications of the procedure and act immediately	◯	◯
Document the indications and events of the procedure in the clinical notes	◯	◯
Communicate the further treatment plan to the nursing staff and patient	◯	◯
Communicate the potential long term complications and failures of the procedure to medical staff and patient	◯	◯

11. If you thought any competencies were inappropriate, please provide some short explanation below

12. Are there additional competencies that you think should be included?

- ◯ No
- ◯ Yes, please add additional competencies to the box below

13. For each of the procedures below, indicate if an intern should be able to perform them at the end of internship

	Yes	No
Venepuncture	◯	◯
Peripheral intravenous cannulation	◯	◯
Blood cultures from a peripheral vein	◯	◯
Arterial Blood Gas Sampling	◯	◯
ECG	◯	◯
Nasogastric tube insertion	◯	◯
Urinary catheter insertion	◯	◯
Prepare, reconstitute, dilute and administer IV drugs	◯	◯
Prime a line	◯	◯
Take blood and blood cultures from a central line	◯	◯
Apply NIV mask and adjust NIV settings	◯	◯
Set up a sterile field	◯	◯
Apply sterile gloves	◯	◯
Perform a sterile surgical scrub and don a surgical gown	◯	◯
Put on and remove PPE	◯	◯

14. If you thought any procedure/s inappropriate, please provide a short explanation below

15. Are there additional procedures that you think should be included?

- ◯ No
- ◯ Yes, please add additional competencies to the box below

EPA 4: Coordinate in-patient care

DESCRIPTION :Upon completion of internship, the doctor should be able to manage their daily workload to prioritise and where necessary delegate tasks, advance patient flow and optimise patient care. They should be able to manage their in-patients' care including requesting investigations and following up the results and generation of flow sheets to formulate a management plan. They should demonstrate leadership skills and be able to lead the team on ward rounds.

16. For each competency, please indicate whether it is appropriate for this EPA.

	Appropriate	Inappropriate
Prepare and consolidate information in advance of ward rounds	○	○
Communicate patient status and data effectively to the team	○	○
Document in clinical notes – SOAP and flow sheets	○	○
Prioritise and delegate tasks	○	○
Review patients and communicate their daily progress plan to staff, family and patient as appropriate	○	○
Lead a ward round	○	○
Request and review laboratory and radiological investigations	○	○
Explain and discuss risks and benefits of common procedures with patients and family members	○	○
Manage challenging patient encounters and situations	○	○
Counsel and educate patients and their families to enable shared decision making	○	○
Develop and carry out patient management plans	○	○
Construct and act on changes in flow sheets	○	○
Manage long-term conditions during episodes of acute care	○	○
Refer to other healthcare providers including physiotherapy, occupational therapy etc where appropriate	○	○
Respond to acute deteriorating patient (EWS)	○	○
Prescribe medication for in-patients considering side effects and interactions	○	○
Perform the necessary procedural skills required as part of in patient care e.g. cannulation, insertion of nasogastric tube	○	○

17. If you thought any competencies were inappropriate, please provide some short explanation below

18. Are there additional competencies that you think should be included?

- ◯ No
- ◯ Yes, please add additional competencies to the box below

EPA 5: Prescribe and monitor drugs and fluids

DESCRIPTION: At the end of internship, the doctor should be able to prescribe safely in both a hospital and community setting – in an elective and emergency setting. They should be able to rationalize and prescribe medicines, blood products and fluids accurately and follow safe prescribing practices (checking expiry dates, double validation and patient identification). They should review patient medications and fluids based on the patient's clinical context.

19. For each competency, please indicate whether it is appropriate for this EPA.

	Appropriate	Inappropriate
Communicate effectively with the ward pharmacist, haemovigilance officer, nursing staff and microbiology department	◯	◯
Take a medication history from patient and other available sources	◯	◯
Discuss drug treatment and administration, including side effects and interactions, with patients	◯	◯
Document medications correctly	◯	◯
Document all allergies and ADRs in the notes and on the prescription	◯	◯
Document the indications for medication changes in the notes	◯	◯
Review medications including appropriate discontinuation or tapering where relevant	◯	◯
Monitor potentially organ toxic drugs (vancomycin, gentamicin)	◯	◯
Request blood products according to best practice and hospital ordering schedules	◯	◯
Manage transfusion reactions	◯	◯
Ensure correct patient identification when prescribing medications, fluids and blood products	◯	◯
Review the requirement for newly started medications based on patients' clinical progress – thrombophylaxis, antibiotics	◯	◯
Use the WHO analgesia ladder to prescribe and step down pain medication	◯	◯
Use and prescribe appropriate oxygen therapies (including NIV)	◯	◯
Recognise , address and inform patient, pharmacy and risk managers of medication errors	◯	◯
Write a discharge prescription	◯	◯
Communicate medication changes to patient and general practitioner	◯	◯

20. If you thought any competencies were inappropriate, please provide some short explanation below

21. Are there additional competencies that you think should be included?

- ◯ No
- ◯ Yes, please add additional competencies to the box below

EPA 6: Recognise and manage the deteriorating/acutely unwell patient.

DESCRIPTION: At the end of internship, the doctor should be able to identify and respond to the acutely unwell patient. They should rapidly determine a working diagnosis based on a focused history and examination, data interpretation, information gathering and situation awareness. They should commence initial management based on clinical reasoning and decision making skills and assess the patient's response to treatment and adapt their management as required. At the same time they should initiate a call for assistance from seniors and escalate or transfer care. They should communicate the situation to medical staff, patient and family members and delegate to nursing staff and colleagues, They should record the clinical encounter in a structured clinical note.

22. For each competency, please indicate whether it is appropriate for this EPA.

	Appropriate	Inappropriate
Recognise the severity of a patient's condition utilising track and trigger systems and situation awareness	◯	◯
Receive a handover from nursing staff and be aware of the patient's recent clinical course	◯	◯
Take a focused history and examination	◯	◯
Determine a working differential diagnosis	◯	◯
Escalate and give a handover on phone or in person	◯	◯
Perform basic technical skills and prescribe as indicated	◯	◯
Apply the principles of standardised protocols as indicated (EWS, ACLS, ATLS, PALS, sepsis 6)	◯	◯
Review the response to initial treatment and respond, act on and adapt treatment as required	◯	◯
Communicate the situation to team members involved in patient care, patient and update family members	◯	◯
Delegate to others	◯	◯
Document the events in the notes	◯	◯
Formulate a further care plan to address prioritised differentials	◯	◯
Plan for transfer as required	◯	◯

23. If you thought any competencies were inappropriate, please provide some short explanation below

24. Are there additional competencies that you think should be included?

- ◯ No
- ◯ Yes, please add additional competencies to the box below

EPA 7: Transition and discharge patient care

DESCRIPTION: By the end of internship, the doctor should be able to hand over and receive the handover of a clinical case to/from a colleague or team (hospital or community based). They should be able to communicate, summarise and document a summary of the patient's clinical course and reason for discharge/transfer.

25. For each competency, please indicate whether it is appropriate for this EPA.

	Appropriate	Inappropriate
Communicate in an open and effective manner with medical staff, patient and family	○	○
Use clinical judgment to recognise the need for transfer/discharge	○	○
Plan in advance for patient discharge and ensure supports are in place to facilitiate this	○	○
Summarise a clinical case verbally, electronically and in the notes	○	○
Prioritise a differential diagnosis	○	○
Formally refer patients to further specialist or tertiary services	○	○
Discharge patients verbally, electronically and in the notes from hospital	○	○
Communicate and liaise with general practitioners and community care services	○	○
Deal with death and follow hospital protocols	○	○
Break bad news to patient and family in a compassionate manner	○	○
Ensure follow up arrangements are in place as appropriate	○	○

26. If you thought any competencies were inappropriate, please provide some short explanation below

27. Are there additional competencies that you think should be included?

- ◯ No
- ◯ Yes, please add additional competencies to the box below

EPA 8: Engage in personal and professional development

DESCRIPTION: At the end of internship, the doctor should have achieved and recorded all of their intern competencies and be a well rounded professional who strives to improve themselves clinically and educationally. They should learn from their mistakes and recognize the learning opportunities that come from feedback. They should have the foresight, motivation and initiative to progress through the intern year and their career to achieve their goals and aspirations in the longer term. Doctors should implement changes to their own practice to improve their performance and patient care. They should advocate and understand the significance of health promotion, and act as role models for other doctors, healthcare professionals and medical students.

28. For each competency, please indicate whether it is appropriate for this EPA.

	Appropriate	Inappropriate
Participate in research - original research, audit, case reports	○	○
Prepare for and present at journal club meetings, grand rounds and national meetings. Look for opportunities to present at international meetings	○	○
Complete and record all education and training requirements (maintains a logbook or e-portfolio) and seek out opportunities to fulfill learning targets	○	○
Engage with undergraduate teaching programmes, teach and contribute to the content of the learning materials and the appraisal of students	○	○
Attend interviews well prepared and with a well constructed up to date curriculum vitae	○	○
Engage with self directed learning – online learning, training days and courses	○	○
Practice in accordance with the Medical Council’s guide to professional conduct and ethics	○	○
Aim to achieve and promote a good work –life balance	○	○
Manages stress and burnout and recognizes it in colleagues	○	○
Is sensitive to different cultures in the workforce	○	○

29. If you thought any competencies were inappropriate, please provide some short explanation below

30. Are there additional competencies that you think should be included?

- ◯ No
- ◯ Yes, please add additional competencies to the box below

EPA 9: Identify compromises to patient care

DESCRIPTION: At the end of the intern year, the doctor should be able to identify compromises in patient care. The doctor should be willing to act as an advocate for the patient. The doctor should be able to report non-adherence to protocol or medical errors in a manner that fosters a just working environment, in which recognition of mistakes lead to learning and improvement. This should include self-reflection and reporting of one's own errors

31. For each competency, please indicate whether it is appropriate for this EPA.

	Appropriate	Inappropriate
Share opinions on patient care	◯	◯
Promote and advocate for patient safety	◯	◯
Respect patient data and confidentiality	◯	◯
Ensure patient privacy when discussing sensitive issues	◯	◯
Identify appropriate forum in which to address concerns (e.g. among team/at hospital admin level…)	◯	◯
Recognise bullying/demeaning behaviours in self/other colleagues	◯	◯
Recognise the symptoms of stress in self/other colleagues	◯	◯
Communicate opinions and concerns assertively without being either too passive or aggressive	◯	◯
Regularly perform honest self-reflection and appraisal of clinical and professional performance	◯	◯
Take appropriate steps to disclose serious incidents or unprofessional practice	◯	◯
Comply with fitness to work statement and occupational health guidelines	◯	◯
Provide care and treatment in accordance with the principles of patients' best interests, autonomy and rights	◯	◯

32. If you thought any competencies were inappropriate, please provide some short explanation below

33. Are there additional competencies that you think should be included?

- ◯ No
- ◯ Yes, please add additional competencies to the box below

34. I am a...

- Intern
- SHO
- Reg
- Consultant

Other (please specify)

35. What is your level of involvement in intern training?

- Network coordinator
- Tutor
- No direct involvement

Other (please specify)

36. Do you have any additional comments?
