# Supplementary material for: The development of a framework of entrustable professional activities for the intern year in Ireland
Source: BMC Med Educ. 2020 Aug 18;20:273. doi: 10.1186/s12909-020-02156-8 (PMC7433170; doi:10.1186/s12909-020-02156-8)
Supplement: Supplementary file 2 — Additional file 2. [file 12909_2020_2156_MOESM2_ESM.pdf]

EPA 1: ADMIT A PATIENT

* 1. Do you agree with the proficiency level for each competency?

- Yes
- No- please provide the competency number, your proposed level of competency, and a brief justification for your answer

* 2. Do you agree with the type of proficiency (i.e. knowledge, skill, or attitude) provided for each competency?

- Yes
- No- please provide the competency number, the type(s) of proficiencies you think are relevant, and a brief explanation for your choice

EPA 2: Request and interpret common investigations

* 3. Do you agree with the proficiency level for each competency?

- ○ Yes
- ○ No- please provide the competency number, your proposed level of competency, and a brief justification for your answer

* 4. Do you agree with the type of proficiency (i.e. knowledge, skill, or attitude) provided for each competency?

- ○ Yes
- ○ No- please provide the competency number, the type(s) of proficiencies you think are relevant, and a brief explanation for your choice

EPA 3: Perform basic procedural skills

* 5. Do you agree with the proficiency level for each competency?

- ◯ Yes
- ◯ No- please provide the competency number, your proposed level of competency, and a brief justification for your answer

* 6. Do you agree with the type of proficiency (i.e. knowledge, skill, or attitude) provided for each competency?

- ◯ Yes
- ◯ No- please provide the competency number, the type(s) of proficiencies you think are relevant, and a brief explanation for your choice

EPA 4: Coordinate in-patient care

* 7. Do you agree with the proficiency level for each competency?

◯ Yes

◯ No- please provide the competency number, your proposed level of competency, and a brief justification for your answer

* 8. Do you agree with the type of proficiency (i.e. knowledge, skill, or attitude) provided for each competency?

◯ Yes

◯ No- please provide the competency number, the type(s) of proficiencies you think are relevant, and a brief explanation for your choice

EPA 5: Prescribe and monitor drugs and fluids

* 9. Do you agree with the proficiency level for each competency?

- ◯ Yes
- ◯ No- please provide the competency number, your proposed level of competency, and a brief justification for your answer

* 10. Do you agree with the type of proficiency (i.e. knowledge, skill, or attitude) provided for each competency?

- ◯ Yes
- ◯ No- please provide the competency number, the type(s) of proficiencies you think are relevant, and a brief explanation for your choice

EPA 6: Recognise and manage the deteriorating/acutely unwell patient.

* 11. Do you agree with the proficiency level for each competency?

- Yes
- No- please provide the competency number, your proposed level of competency, and a brief justification for your answer

* 12. Do you agree with the type of proficiency (i.e. knowledge, skill, or attitude) provided for each competency?

- Yes
- No- please provide the competency number, the type(s) of proficiencies you think are relevant, and a brief explanation for your choice

EPA 7:

* 13. Do you agree with the proficiency level for each competency?

◯ Yes

◯ No- please provide the competency number, your proposed level of competency, and a brief justification for your answer

* 14. Do you agree with the type of proficiency (i.e. knowledge, skill, or attitude) provided for each competency?

◯ Yes

◯ No- please provide the competency number, the type(s) of proficiencies you think are relevant, and a brief explanation for your choice

EPA 8: Engage in personal and professional development

* 15. Do you agree with the proficiency level for each competency?

- Yes
- No- please provide the competency number, your proposed level of competency, and a brief justification for your answer

* 16. Do you agree with the type of proficiency (i.e. knowledge, skill, or attitude) provided for each competency?

- Yes
- No- please provide the competency number, the type(s) of proficiencies you think are relevant, and a brief explanation for your choice

EPA 9: Identify compromises to patients’ care

* 17. Do you agree with the proficiency level for each competency?

- Yes
- No- please provide the competency number, your proposed level of competency, and a brief justification for your answer

* 18. Do you agree with the type of proficiency (i.e. knowledge, skill, or attitude) provided for each competency?

- Yes
- No- please provide the competency number, the type(s) of proficiencies you think are relevant, and a brief explanation for your choice

* 19. I am a...

- Intern
- SHO
- Reg
- Consultant

Other (please specify)

* 20. What is your level of involvement in intern training?

- Network coordinator
- Tutor
- No direct involvement

Other (please specify)
