# Supplementary material for: The development of a framework of entrustable professional activities for the intern year in Ireland
Source: BMC Med Educ. 2020 Aug 18;20:273. doi: 10.1186/s12909-020-02156-8 (PMC7433170; doi:10.1186/s12909-020-02156-8)
Supplement: Supplementary file 3 — Additional file 3. [file 12909_2020_2156_MOESM3_ESM.docx]

**Additional File 3: Medical council consultation Survey questions**

| **Question** |
| --- |
| What aspects of intern training do you think are working well at the moment? |
| What aspects of intern training do you think  could be improved? |
| Do you agree that defined competencies should be established for successful completion of intern training and award of a certificate of experience? |
| Do you agree with an EPA approach being used to describe what doctors are expected to achieve at the end of intern training? |
| Do you agree the following should be an EPA? |
| Before making a final decision on the proposed EPA framework, the Medical Council now invites comments on what you think this will mean for intern training in Ireland |
