# Supplementary material for: The development of a framework of entrustable professional activities for the intern year in Ireland
Source: BMC Med Educ. 2020 Aug 18;20:273. doi: 10.1186/s12909-020-02156-8 (PMC7433170; doi:10.1186/s12909-020-02156-8)
Supplement: Supplementary file 4 — Additional file 4. [file 12909_2020_2156_MOESM4_ESM.docx]

**Additional File 4: EPA Template**

|  | **EPA No.** |  |
| --- | --- | --- |
| **A.** | **Title** |  |
| **B.** | **Prerequisites** |  |
| **C.** | **Description of the activity** |  |
| **D.** | **Alignment with Medical Council Domains and Pillars** |  |
| **E.** | **Proficiency** |  |
| **F.** | **Competencies** | |
|  | *Add rows* | |
| **G.** | **Observation and Review tool/s**  (indicated) | Case Presentation (CP)  Direct Observation of Procedural Skills (DOPS)  Case Based Discussion (CBD)  Reflective Journal (RJ)  Team Review (TR) – used across EPAs |
| **H.** | **Basis for entrustment at level 4** | Number/range of successfully completed observations/reviews.  These provide the ‘basis for entrustment’ to perform the activity at level 4, i.e. independently with mainly informal, indirect supervision, where assessor was on-site and available, just in case. |
| **I.** | **Volume of practice** | Minimum volume of practice required with a guide per year (Recorded in the Intern Log and signed by supervisor). |

**Key to levels**

| **Level** | **Proficiency - level of ‘entrustability’** |
| --- | --- |
| **1** | Intern has acquired relevant knowledge and skills, but not enough to perform the activity. |
| **2** | Intern may perform an activity under direct supervision, with supervisor in the same room, deciding the intensity of supervision required. |
| **3** | The intern may perform an activity with direct, intermittent supervision: the intern asks for supervision as required. |
| **4** | The intern may perform an activity independently with mainly informal, indirect supervision. |
| **5** | Intern may provide supervision and instruction to junior learners |
