# Supplementary material for: The development of a framework of entrustable professional activities for the intern year in Ireland
Source: BMC Med Educ. 2020 Aug 18;20:273. doi: 10.1186/s12909-020-02156-8 (PMC7433170; doi:10.1186/s12909-020-02156-8)
Supplement: Supplementary file 5 — Additional file 5. [file 12909_2020_2156_MOESM5_ESM.docx]

**Additional File 5: Table of Agreement for stages 4 and 5.**

| **Stage 4** | | |
| --- | --- | --- |
| Draft EPA | % agree that EPA is a core requirement | % of competencies which >90% of respondents agreed should be performed by an intern |
| Admit a patient | 90.9% | 85.2% |
| Request and interpret investigations | 100% | 100% |
| Perform basic procedural skills | 100% | 81.4% |
| Manage the work of in-patient care | 100% | 88.2% |
| Prescribe and monitor drugs and fluid | 100% | 82.3% |
| Recognise and manage the deteriorating/acutely unwell patient | 100% | 84.6% |
| Transition and discharge patient care | 90.9% | 54.5% |
| Engage in personal and professional development | 100% | 100% |
| Identify compromises to patient care | 100% | 100% |
| **Stage 5** | | |
| Draft EPA | % of respondents who agreed with all of the proficiency ratings of the competencies in the EPA | % of respondents who agreed with the classification of the type of competency in each EPA |
| Admit a patient | 83.3% | 58.3% |
| Request and interpret investigations | 90.9% | 90.9% |
| Perform basic procedural skills | 72.7% | 81.8% |
| Manage the work of in-patient care | 70.0% | 90% |
| Prescribe and monitor drugs and fluid | 90% | 90% |
| Recognise and manage the deteriorating/acutely unwell patient | 90% | 90% |
| Transition and discharge patient care | 100% | 90% |
| Engage in personal and professional development | 90% | 90% |
| Identify compromises to patient care | 80% | 90% |
