# Supplementary material for: The development of a framework of entrustable professional activities for the intern year in Ireland
Source: BMC Med Educ. 2020 Aug 18;20:273. doi: 10.1186/s12909-020-02156-8 (PMC7433170; doi:10.1186/s12909-020-02156-8)
Supplement: Supplementary file 6 — Additional file 6. [file 12909_2020_2156_MOESM6_ESM.docx]

**Additional File 6: Intern Year EPAs and their linked competencies.**

|  | **EPA** | **No. 1** | | |
| --- | --- | --- | --- | --- |
| **A.** | **Title** | **Clerk a patient** | | |
| **B.** | **Prerequisites** | Meet eligibility criteria for internship as determined by the Health Service Executive (HSE), National Recruitment Service (NRS) & Medical Council (MC)  Basic Life Support (BLS) Certification | | |
| **C.** | **Description of the activity** | At the end of internship, the doctor is able to clerk a patient in the outpatient and day case setting, admit a patient to the ward (elective, emergency) and have a good understanding of decision to admit criteria. The doctor can communicate effectively and establish a partnership with the patient and their relatives. The clerking or admission should record a focused history, a thorough physical examination and a record of any pathological findings. This should form the basis for requesting laboratory and radiological investigations and consultations that are pertinent to the case, rationalised and reflect best practice. The clinical note/document should be logically structured, and the doctor should be able to prioritise diagnoses, interpret investigations and formulate a treatment plan. They should communicate a summarized account to colleagues and healthcare professionals. Recognition of complex cases should happen early, and the doctor should seek expert and more senior help immediately. | | |
| **D.** | **MC domains/pillars of professionalism** | Domain 2, Relating to Patients; Domain 3, Communication and Interpersonal Skills; Domain 7, Clinical Skills; Pillar (i), Professionalism: Partnership; Pillar (ii), Professionalism: Practice. | | |
| **E.** | **Proficiency** | **Level 4 (i.e. the intern may perform an activity independently with mainly informal, indirect supervision)** | | |
| **F.** | **Competencies** | | **Tools** | |
|  |  |  | **CP** | **CBD** |
| 1 | Establish rapport with the patient | | **X** |  |
| 2 | Consider factors that may affect the patient’s capacity to describe their symptoms or understand questions and/or give informed consent | | **X** | **X** |
| 3 | Take a focused history in a range of contexts and conditions | | **X** | **X** |
| 4 | Recognise complex cases and seek help | |  | **X** |
| 5 | Obtain a history from other sources as required (e.g. collateral, own doctor, pharmacy, clinical notes) | | **X** | **X** |
| 6 | Share patient information only as appropriate with relevant parties | |  | **X** |
| 7 | Perform a fluid and sequential clinical examination | | **X** |  |
| 8 | Demonstrate respect for the patient’s privacy, dignity and culture | | **X** |  |
| 9 | Identify abnormal clinical findings | | **X** | **X** |
| 10 | Request laboratory and radiological investigations and consultations that are pertinent to the case and reflect best practice | | **X** | **X** |
| 11 | Act on conditions and presentations that require immediate intervention, instigate initial resuscitation/treatment and call for senior help | | **X** |  |
| 12 | Formulate a differential diagnosis | | **X** | **X** |
| 13 | Devise a treatment and further management plan for review by senior clinician | | **X** | **X** |
| 14 | Document all findings in the patient chart to comply with good professional practice | |  | **X** |
| 15 | Follow prescribing protocols and discuss medications with senior colleagues | | **X** | **X** |
| 16 | Recognise patients at risk of deterioration | |  | **X** |
| 17 | Communicate with senior colleagues and nursing staff to ensure all have a shared mental model of the patient’s condition and needs | |  | **X** |
| 18 | Communicate openly and honestly with families and relatives of patients | | **X** | **X** |
| **G.** | **Observation and Review tool/s** | **Case presentation (CP)**  **Case based discussion (CBD)** | | |
| **H.** | **Basis for entrustment** | **Successful completion of the all of following at level 4** | **CP** | **CBD** |
|  |  | General medical case | **1** |  |
|  |  | General surgical case | **1** |  |
|  |  | Specialist case (e.g. paediatrics, obstetrics and gynaecology, orthopaedics, cardiology) | **2** |  |
|  |  | Complex case | **1** |  |
|  |  | Any case |  | **1** |
| **I.** | **Volume of practice** | Minimum of 40 patients clerked in the year (recorded in the intern’s log) - a guide average of 10 per rotation | | |

|  | **EPA** | **No. 2** | | |
| --- | --- | --- | --- | --- |
| **A.** | **Title** | **Request and interpret basic investigations** | | |
| **B.** | **Prerequisites** | Meet eligibility criteria for internship as determined by the Health Service Executive (HSE), National Recruitment Service (NRS) & Medical Council (MC)  Basic Life Support (BLS) Certification | | |
| **C.** | **Description of the activity** | At the end of internship, the doctor can request appropriate and interpret basic diagnostic laboratory and radiological investigations (plain films, basic haematology, biochemistry & microbiology panels for example). They can explain and rationalise the requirement for investigations to the patient and medical team and communicate the indications to the relevant laboratories and departments.  Their interpretation of these investigations should reflect the clinical context and lead to formulation of a most likely diagnosis and prioritisation of differential diagnoses. The doctor should use the results to treat the patient and/or to inform the need for further investigations. | | |
| **D.** | **MC domains/pillars of professionalism** | Domain 1, Patient Safety and Quality of Patient Care; Domain 6, Scholarship; Domain 7, Clinical Skills; Pillar (ii), Professionalism: Practice; P(iii), Professionalism: Performance | | |
| **E.** | **Expected proficiency** | **Level 4 (i.e. the intern may perform an activity independently with mainly informal, indirect supervision)** | | |
| **F.** | **Competencies** | | **Tools** | |
|  |  |  | **CP** | **CBD** |
| 1 | Request investigations and screening tests based on patient information, best practice and team consultation | | **X** | **X** |
| 2 | Work together with patients so they can be involved in the decision-making process | |  | **X** |
| 3 | Correctly identify the patient and patient details so as to prevent error | |  | **X** |
| 4 | Factor in cost-effectiveness, risk-benefit analysis to the choice of investigation | |  | **X** |
| 5 | Communicate the clinical situation and rationale for the investigation to laboratory, radiology or other departments | |  | **X** |
| 6 | Actively seek the result of the investigation, including follow through on the request, as necessary | |  | **X** |
| 7 | Interpret the results of basic radiology reports and images and basic laboratory reports required for the area of practice | | **X** | **X** |
| 8 | Record the results of investigations in patient notes and the implications of the results in the patient’s further care and management plan | |  | **X** |
| 9 | Communicate results and their meaning to the medical team | | **X** | **X** |
| 10 | Explain investigation results and their meaning to the patient and/or family, other than for cases requiring senior clinician involvement. | | **X** | **X** |
| 11 | Identify further investigations or screening as required | |  | **X** |
| 12 | Act immediately on abnormal results and seek assistance as appropriate | |  | **X** |
| **G.** | **Observation and Review tool/s** | **Case presentation (CP)**  **Case based discussion (CBD)** | | |
| **H.** | **Basis for entrustment** | **Successful completion of the all of following at level 4** | **CP** | **CBD** |
|  |  | General medical case |  | **1** |
|  |  | General surgical case |  | **1** |
|  |  | Specialist case (e.g. paediatrics, obstetrics and gynaecology, orthopaedics, cardiology) |  | **2** |
|  |  | Complex case | **1** |  |
|  |  | Any case | **1** |  |
| **I.** | **Volume of practice** | Minimum of 40 requests for investigations (and interpretation) per year (recorded in the intern’s log) – guide average of 10 per rotation | | |

|  | **EPA** | **No. 3** | |
| --- | --- | --- | --- |
| **A.** | **Title** | **Perform essential procedural skills** | |
| **B.** | **Prerequisites** | Meet eligibility criteria for internship as determined by the Health Service Executive (HSE), National Recruitment Service (NRS) & Medical Council (MC)  Basic Life Support (BLS) Certification | |
| **C.** | **Description of the activity** | By the end of internship, the doctor demonstrates confident and is skilled in performing **all** of the following essential procedures:   - Hand hygiene - Venipuncture - Peripheral intravenous cannulation - Blood cultures from a peripheral vein - Arterial blood gas sampling - Electrocardiogram (ECG) - Nasogastric tube insertion - Urinary catheter insertion - Preparation, reconstitution, dilution and administeration of iv drugs - Blood sampling and blood cultures from central line and tunelled lines - Sterile field set up - Sterile glove application   Other “non - essential” procedures – see list in Appendix G | |
| **D.** | **MC domains/pillars of professionalism** | Domain 2, Relating to patients; Domain 7, Clinical skills; Pillar (iii), Professionalism: performance | |
| **E.** | **Expected proficiency** | **Level 4 (i.e. the intern may perform an activity independently with mainly informal, indirect supervision)** | |
| **F.** | **Competencies** | | **Tools** |
|  |  |  | **DOPS** |
| 1 | Describe the indications and contraindictions for a procedure | | **X** |
| 2 | Perform procedures cost - effectively, based on patient information and preferences and maintaining patient privacy | | **X** |
| 3 | Recognise own limitations in potentially complex patient cases and call for help | | **X** |
| 4 | Perform 5 moments of hand hygiene | | **X** |
| 5 | Use aseptic non-touch technique | | **X** |
| 6 | Obtain verbal informed consent from the patient by explaining the indications, alternatives, potential complications and implications of failure of the procedure | | **X** |
| 7 | Put the patient at ease and establish trust | | **X** |
| 9 | Adopt standard operating procedures and national standards for clinical practice guidelines | | **X** |
| 10 | Set up a sterile field, maintain aseptic and sterile conditions | | **X** |
| 11 | Dispose of hazardous and clinical waste as per protocols | | **X** |
| 12 | Recognise any immediate failures or complications of the procedure and act immediately | | **X** |
| 13 | Document the indications and events of the procedure in the clinical notes | | **X** |
| 14 | Communicate the further treatment plan to the nursing staff and patient | | **X** |
| **G** | **Observation and Review tool/s** | Direct Observation of Procedural Skills (DOPS) | |
| **H.** | **Basis for entrustment** | **Successful completion of the all of following at level 4** | **DOPS** |
|  |  | 1 DOPS per essential procedure | **12** |
| **I.** | **Volume of practice** | Minimum of 10 of each of the essential procedures per year (recorded in the intern’s log - 120 in total) – a guide average of 30 per rotation  Evidence of experience in 2 non-essential procedures | |

|  | **EPA** | **No. 4** | | | |
| --- | --- | --- | --- | --- | --- |
| **A.** | **Title** | **Manage the work of in-patient care** | | | |
| **B.** | **Prerequisites** | Meet eligibility criteria for internship as determined by the Health Service Executive (HSE), National Recruitment Service (NRS) & Medical Council (MC) Basic Life Support (BLS) Certification | | | |
| **C.** | **Description of the activity** | Upon completion of internship, the doctor can manage their daily workload to prioritise and, where necessary, delegate tasks, advance patient flow and deliver patient centered care by implementing clinical practice guidelines. They can manage their in-patients’ care including requesting investigations and following up the results and generating of flow sheets to formulate a management plan. They can use written and electronic communication forms. They are able to work with teams and colleagues and communicate by listening to and sharing information and advice. They know their limitations and seek senior input and advice when appropriate. They communicate with families and relatives in an open and honest manner to give information and explanations. | | | |
| **D.** | **MC domains/pillars of professionalism** | Domain 4, Collboration and Teamwork; Domain 5, Management (Including Self Management); Pillar (i), Professionalism: Partnership; Pillar (ii), Professionalism: Practice; Pillar(iii), Professionalism: Performance | | | |
| **E.** | **Expected proficiency** | **Level 4 (i.e. the intern may perform an activity independently with mainly informal, indirect supervision)** | | | |
| **F.** | **Competencies** | | **Tools** | | |
|  |  |  | **CP** | **CBD** | **RJ** |
| 1 | Prepare and consolidate information in advance of ward rounds and theatre/ procedure lists | | **X** | **X** |  |
| 2 | Communicate patient status and data effectively to the team | | **X** |  |  |
| 3 | Keep good records in clinical notes – SOAP and flow sheets | |  | **X** |  |
| 4 | Prioritise and delegate tasks | |  | **X** | **X** |
| 5 | Contribute to patient management and recognise cases or situations requiring senior  clinician involvement | |  | **X** | **X** |
| 6 | Review patients and communicate their daily progress plan to healthcare staff | | **X** |  |  |
| 7 | Review, record and report trends and changes in laboratory and radiological investigations | | **X** | **X** |  |
| 8 | Respond to requests from team and nursing staff in a timely manner | |  | **X** |  |
| 9 | Consent patients on behalf of a senior doctor and in line with MC guidelines, and appreciate the extent of a patient’s autonomy over healthcare decisions | | **X** |  |  |
| 10 | Contribute to the management of long-term conditions during episodes of acute care | |  |  | **X** |
| 11 | Take account of the input and views of other healthcare providers’ views, knowledge, skills and experiences to work as an effective team | |  | **X** |  |
| 12 | Respond to acutely deteriorating patient (NEWS) | |  | **X** | **X** |
| 13 | Identify their level of clinical experience to patients and family when discussing patient care and defer to more a more senior clinician | |  | **X** |  |
| 14 | Manage challenging patient encounters and situations with senior support | |  |  | **X** |
| 15 | Pronounce death of patients and follow post mortem protocols | |  | **X** | **X** |
| 16 | Discuss options with patients and their families to enable shared decision making, other than for cases requiring senior clinician involvement | | **X** |  | **X** |
| 17 | Deal with patient issues in a sensitive and non-judgmental manner | | **X** | **X** | **X** |
| 18 | Participate in breaking of bad news to patient and family in a compassionate manner, with a senior clinician | |  |  | **X** |
| 19 | Disclose serious and adverse events or harm done in the course of healthcare to patients and families, for procedures for which they are responsible and know the reporting mechanisms | |  | **X** | **X** |
| 20 | Maintain patient data, confidentiality and trust | |  | **X** | **X** |
| **G.** | **Observation and Review tool/s** | **Case presentation (CP)**  **Case based discussion (CBD)**  **Reflective journal (RJ)** | | | |
| **H.** | **Basis for entrustment at level 4** | **Successful completion of the all of following at level 4** | **CP** | **CBD** | **RJ** |
|  |  | General medical case | **1** | **1** | **3** |
|  |  | General surgical case | **1** | **1** |  |
|  |  | Specialist case (e.g. paediatrics, obstetrics and gynaecology, orthopaedics, cardiology) | **1** | **1** |  |
|  |  | Complex case | **1** | **1** |  |
|  |  | Any case | **1** | **1** |  |
| **I.** | **Volume of practice** | Intern needs to manage the care of a minimum of 40 patients per year - a guide average of 10 per rotation (recorded in the intern’s log) | | | |

|  | **EPA** | **No. 5** | | |
| --- | --- | --- | --- | --- |
| **A.** | **Title** | **Prescribe and monitor drugs and fluids** | | |
| **B.** | **Prerequisites** | Meet eligibility criteria for internship as determined by the Health Service Executive (HSE), National Recruitment Service (NRS) & Medical Council (MC)  Basic Life Support (BLS) Certification | | |
| **C.** | **Description of the activity** | At the end of internship, the doctor is able to prescribe safely in compliance with legal requirements, in both a hospital and community setting and in an elective and emergency setting. They can rationalize and prescribe medicines, blood products, oxygen and fluids accurately and follow safe prescribing practices (checking expiry dates, double validation and patient identification). They are able to review and prescribe patient medications and fluids based on assessment of the patient and in the context of the patient’s clinical status (for example, age weight, renal function, hydration status, cardiac status etc.) | | |
| **D.** | **MC domains/pillars of professionalism** | Domain 1, Patient Safety and Quality of Patient Care; Domain 6, Scholarship; Domain 7, Clinical Skills; Pillar (ii), Professionalism: Practice; P(iii), Professionalism: Performance | | |
| **E.** | **Expected proficiency** | **Level 4 (i.e. the intern may perform an activity independently with mainly informal, indirect supervision)** | | |
| **F.** | **Competencies** | | **Tools** | |
|  |  |  | **CP** | **CBD** |
| 1 | Communicate effectively with the ward pharmacist, haemovigilance officer, nursing  staff and microbiology department | |  | **X** |
| 2 | Take a medication history from patient and other available sources | | **X** | **X** |
| 3 | Adjust medication and fluid doses according to age, weight, height, renal function,  cardiac function etc. in consultation with senior clinician | | **X** | **X** |
| 4 | Explain drug, blood and fluid treatment and administration, to patients, including side effects and interactions | | **X** | **X** |
| 5 | Prescribe blood, fluids and medications legibly and correctly | |  | **X** |
| 6 | Consult medication guidelines when reconstituting drugs | |  | **X** |
| 7 | Document all allergies and ADRs in the notes and on the prescription | |  | **X** |
| 8 | Manage anaphylaxis with a senior clinician | |  | **X** |
| 9 | Document medication changes and the indications for medication changes in the clinical notes | |  | **X** |
| 10 | Review medications including appropriate discontinuation or tapering where relevant (for example - IV to Oral switch) in consultation with senior clinician | | **X** | **X** |
| 11 | Prescribe and administer fluids and blood having elicited symptoms and signs of clinical need (e.g. dehydration, anaemia) | |  | **X** |
| 12 | Recognise the need to monitor potentially organ toxic drugs (vancomycin, gentamicin) | | **X** | **X** |
| 13 | Request blood products according to best practice and hospital ordering schedules | | **X** | **X** |
| 14 | Manage common transfusion reactions | |  | **X** |
| 15 | Ensure correct patient identification when prescribing medications, fluids and blood products | | **X** | **X** |
| 16 | Review the requirement for newly started medications based on patients’ clinical progress – thromboprophylaxis, antibiotics, in consultation with senior clinician | | **X** | **X** |
| 17 | Use the WHO analgesia ladder to prescribe and step down pain medication, in consultation with senior clinician | | **X** | **X** |
| 18 | Use and prescribe appropriate oxygen therapies and review response | | **X** | **X** |
| 19 | Recognise, address and inform patient, pharmacy and risk managers of medication errors | |  | **X** |
| 20 | Write or complete an electronic discharge prescription including standard, non-technical, technical and MDA prescriptions | |  | **X** |
| 21 | Communicate medication changes to patient, family and general practitioner | | **X** | **X** |
| **G.** | **Observation and Review tool/s** | **Case presentation (CP)**  **Case based discussion (CBD)** |  |  |
| **H.** | **Basis for entrustment at level 4** | **Successful completion of the all of following at level 4** | **CP** | **CBD** |
|  |  | General medical case | **1** | **1** |
|  |  | General surgical case | **1** | **1** |
|  |  | Specialist case (e.g. paediatrics, obstetrics and gynaecology, orthopaedics, cardiology) | **1** | **1** |
|  |  | Complex case |  | **1** |
|  |  | Any case |  | **1** |
| **I.** | **Volume of practice** | Intern needs to prescribe (and monitor) for 40 cases per year - a guide average of 10 per rotation (recorded in the intern’s log) | | |

|  | **EPA** | **No. 6** | | | |
| --- | --- | --- | --- | --- | --- |
| **A.** | **Title** | **Recognise and manage the deteriorating/acutely unwell patient** | | | |
| **B.** | **Prerequisites** | Meet eligibility criteria for internship as determined by the Health Service Executive (HSE), National Recruitment Service (NRS) & Medical Council (MC)  Basic Life Support (BLS) Certification | | | |
| **C.** | **Description of the activity** | At the end of internship, the doctor identifies and responds to the acutely unwell patient. They rapidly determine a working diagnosis based on a focused history and examination, data interpretation, information gathering and situation awareness. They commence initial management based on clinical reasoning and decision-making skills and assess the patient’s response to treatment and adapt their management as required. At the same time they should recognise their limitations and initiate a call for assistance from seniors and escalate or recognise the need to transfer care. They effectively communicate the situation to medical staff, patient and family members and be able to delegate to nursing staff and colleagues. They are able to review and reflect with their team on the outcomes of acute care cases and keep up to date with international practice and guidelines (for example ACLS and sepsis six guidelines). | | | |
| **D** | **MC domains/pillars** | Domain 1, Patient Safety and Quality of Patient Care; Domain 4, Collaboration and Teamwork; Domain 7, Clinical Skills; Pillar (i), Professionalism: Partnership | | | |
| **E.** | **Expected proficiency** | **Level 4 (i.e. the intern may perform an activity independently with mainly informal, indirect supervision)** | | | |
| **F.** | **Competencies** | | **Tools** | | |
|  |  |  | **CP** | **CBD** | **RJ** |
| 1 | Recognise the severity of a patient’s condition utilising track and trigger systems and situation awareness | | **X** | **X** | **X** |
| 2 | Receive a handover from nursing staff and be aware of the patient’s recent clinical course | | **X** |  |  |
| 3 | Take a focused history and examination | | **X** | **X** |  |
| 4 | Determine a working differential diagnosis | | **X** | **X** |  |
| 5 | Call for help from senior clinicians, escalate care and give a handover on phone or in person | |  | **X** | **X** |
| 6 | Apply the principles of standardised protocols and clinical guidelines as indicated (NEWS, ACLS, ATLS, PALS, sepsis six bundles) | | **X** | **X** |  |
| 7 | Review patient response to initial treatment and respond, act on and adapt treatment as required, in consultation with senior clinician | |  | **X** | **X** |
| 8 | Communicate the situation to team members involved in patient care, patient and update family members, with a senior clinician | |  | **X** | **X** |
| 9 | Delegate to others in the team | |  | **X** | **X** |
| 10 | Document the events in the clinical notes in a well structured format | |  | **X** |  |
| 11 | Formulate a further care plan to address prioritised differentials with a senior clinician | | **X** | **X** |  |
| 12 | Participate in breaking of bad news to patient and family in a compassionate manner, with a senior clinician | |  | **X** | **X** |
| 13 | Debrief with the team and review the outcomes | |  |  | **X** |
| 14 | Reflect on outcomes and performance and identify areas for improvement | |  |  | **X** |
| **G.** | **Observation and Review tool/s** | **Case based discussion (CBD)**  **Case presentation (CP)**  **Reflective journal (RJ)** |  |  |  |
| **H.** | **Basis for entrustment at level 4** | **Successful completion of the all of following at level 4** | **CP** | **CBD** | **RJ** |
|  |  | General medical case | **1** | **1** | **3** |
|  |  | General surgical case | **1** | **1** |  |
|  |  | Specialist case (e.g. paediatrics, obstetrics and gynaecology, orthopaedics, cardiology) | **1** | **1** |  |
|  |  | Complex case |  | **1** |  |
|  |  | Any case |  | **1** |  |
| **I.** | **Volume of practice** | Intern needs to recognise (and manage) 20 cases - a guide average of 5 per rotation (recorded in the intern’s log) | | | |

|  | **EPA** | **No. 7** | | | |
| --- | --- | --- | --- | --- | --- |
| **A.** | **Title** | **Handover and discharge a patient** | | | |
| **B.** | **Prerequisites** | Meet eligibility criteria for internship as determined by the Health Service Executive (HSE), National Recruitment Service (NRS) & Medical Council (MC)  Basic Life Support (BLS) Certification | | | |
| **C.** | **Description of the activity** | By the end of internship, the doctor can handover and receive the handover of a clinical case to/from a healthcare worker colleague or team (hospital or community based). They communicate, summarise and document a review of the patient’s clinical course and highlight the changes made to previous medications and treatment plans. They can explain the reason for discharge/transfer to receiving clinician, family and patient and ensure all up to date records and results are available to the receiving clinician. | | | |
| **D.** | **MC domains/pillars of professionalism** | Domain 2, Relating to patients; Domain 3, Communication and Interpersonal Skills; Domain 4, Collaboration and Teamwork; Pillar (i), Professionalism: Partnership; Pillar (ii), Professionalism: Practice | | | |
| **E.** | **Expected proficiency** | **Level 4 (i.e. the intern may perform an activity independently with mainly informal, indirect supervision)** | | | |
| **F.** | **Competencies** | | **Tools** | | |
|  |  |  | **CP** | **CBD** | **RJ** |
| 1 | Communicate the clinical situation in an open, honest and effective manner with  medical staff, patient and family | |  |  | **X** |
| 2 | Use clinical judgment to recognise the need for handover or transfer/discharge | |  | **X** | **X** |
| 3 | Perform a fluid handover of patients at end of and change of shifts and | |  |  |  |
| 4 | Plan in advance for patient discharge and ensure supports are in place to facilitate discharge (for example home supports and assistance) | |  | **X** |  |
| 5 | Summarise a clinical case verbally, electronically/in writing in the clinical notes using a  structured system such as ISBAR when handing over patient care | | **X** | **X** |  |
| 6 | Perform a fluid handover of patients at end of and change of shifts at the appointed time and  place | | **X** | **X** |  |
| 7 | Record a diagnosis as well as active and inactive secondary diagnoses accurately | |  | **X** |  |
| 8 | Formally refer patients to further specialist or tertiary services | |  | **X** |  |
| 9 | Determine patient fitness to work (and return to work) | | **X** | **X** | **X** |
| 10 | Participate in transfer of patient to another healthcare receiver (ward, theatre, ICU, GP) and/or family. | |  | **X** | **X** |
| 11 | Discharge patients verbally, electronically and in the notes from hospital | |  | **X** |  |
| 12 | Ensure follow up arrangements are in place as appropriate | |  | **X** |  |
| **G.** | **Observation and Review tool/s** | **Case based discussion (CBD)**  **Case presentation (CP)**  **Reflective journal (RJ)** |  |  |  |
| **H.** | **Basis for entrustment at level 4** | **Successful completion of the all of following at level 4** | **CP** | **CBD** | **RJ** |
|  |  | General medical case | **1** | **1** | **3** |
|  |  | General surgical case | **1** | **1** |  |
|  |  | Specialist case (e.g. paediatrics, obstetrics and gynaecology, orthopaedics, cardiology) | **1** | **1** |  |
|  |  | Complex case |  | **1** |  |
|  |  | Any case |  | **1** |  |
| **I.** | **Volume of practice** | Intern needs to participate in handover or discharge of 40 patients - a guide average of 10 per rotation (recorded in the intern’s log) | | | |

|  | **Set of related activities** |  |  |
| --- | --- | --- | --- |
| **A.** | **Title** | **Engage in personal and professional development (PPD)** |  |
| **B.** | **Prerequisites** | Meet eligibility criteria for internship as determined by the Health Service Executive (HSE), National Recruitment Service (NRS) & Medical Council (MC)  Basic Life Support (BLS) Certification |  |
| **C.** | **Description of the activity** | At the end of internship, the doctor has achieved all EPAs to a level 4, and be a well-rounded professional who strives to improve themselves clinically and educationally. They are aware of their limitations. They are capable of learning from mistakes and recognise the learning opportunities that come from feedback. They actively seek out learning and feedback opportunities.  They have the foresight, motivation and initiative to focus on achieving longer term training opportunities and goals. They support and participate in health promotion, audit and research.  They advocate for patients and strive to maintain patient trust in themselves and the medical profession. They act as role models for other doctors, healthcare professionals and medical students. |  |
| **D.** | **MC domains/pillars of professionalism** | Domain 1, Patient Safety and Quality of Patient Care; Domain 6, Scholarship; Pillar (ii), Professionalism: Practice; P(iii), Professionalism: Performance |  |
| **E.** | **Expected proficiency** | **Level 4 (i.e. the intern may perform an activity independently with mainly informal, indirect supervision)** |  |
| **F.** | **Competencies** | | |
|  |  |  |  |
| 1 | Participate in research – basic original research, case reports etc. | | |
| 2 | Participate in a clinical audit | | |
